# The Impact of Support Intensity Needs on Person-Centred Case Management

**DOI:** 10.3390/healthcare13212697

**Published:** 2025-10-25

**Authors:** Paolo Bianchi, Marco Lombardi, Luigi Croce, Antonio Caserta, Roberta Speziale

**Affiliations:** 1 Nottingham University Business School China, University of Nottingham Ningbo China, Ningbo 315100, China; 2 EQUALTY Research Collective, HOGENT, University of Applied Sciences and Arts, 9000 Ghent, Belgium; marco.lombardi@hogent.be; 3 Dipartimento di Pedagogia, Università Cattolica del Sacro Cuore, 25121 Brescia, Italy; luigi.croce@unicatt.it; 4 ANFFAS, 00179 Rome, Italy; acaserta@anffas.net (A.C.); roberta.s@anffas.net (R.S.)

**Keywords:** intellectual and developmental disability, person-centred care, resource use, support needs

## Abstract

**Background:** International and national policies increasingly call for person-centred approaches in disability services, yet little is known about how support intensity needs influence the allocation of resources for individuals with intellectual and developmental disabilities (IDDs). In Italy, where integrated socio-health systems operate within a human rights framework, this quantitative study investigates how individual and contextual factors shape resource use in individualized support planning. **Methods:** We analyzed data from 1152 adults with IDDs enrolled in 23 service centres across 13 Italian regions. Case managers developed Individualized Support Plans (ISPs) informed by the Supports Intensity Scale and socio-ecological variables. Resource use was measured as weekly counts of adaptive skills training, community participation supports, habilitation services, prosthetics, and assistive technologies. We applied multivariate count models (Sarmanov–Lee) to capture the interdependence across support types. **Results:** Findings show that gender and level of intellectual functioning did not significantly affect resource allocation. However, individuals with the highest support intensity needs often received fewer supports, particularly in adaptive skills and community participation. Residential settings were associated with higher levels of support provision compared to family or independent living. Assistive technologies and prosthetics were linked with more comprehensive support packages. **Conclusions:** While person-centred planning frameworks are being implemented, systemic inequities remain, with those at the highest levels of need at risk of receiving fewer enabling supports. Multivariate modelling provides a robust tool for understanding resource use and highlights the importance of equity-focused planning. These findings support policy and practice reforms that operationalize human rights principles and align with the UNCRPD, ensuring more inclusive and responsive systems of support.

## 1. Introduction

The sector of social care and related healthcare services for people with intellectual and developmental disabilities (IDDs) has experienced a paradigmatic transition in the last few decades. The gradual shift from the medical model and the growing acceptance of the socio-ecological model of disability and human rights have accompanied the emergence of a person-centred approach, where assistance and support plans aim to overcome individual-specific disabling barriers [1,2], to fulfil the empowerment of the person with a disability, warrant independent life, and the enjoyment of human rights [3,4].

The main aim of this study was to investigate the impact of socio-ecological factors on resource use in person-centred case management for adults with IDDs. The associated aim was to evaluate a flexible multivariate approach to model the pattern of resource use in complex care, contributing to the debate on the comparative usefulness between univariate and multivariate approaches in such a context [5]. In contrast to previous research based on single case studies, we used anonymized individual data from a large national study [6]. Our sample included both individuals residing in service communities and individuals living within their family households; the former group has been usually excluded from traditional statistical surveys and empirical studies [7].

In Italy, care services for people with IDDs are provided within an integrated socio-health system. The Italian disability services sector is characterized by the presence of both public and private organizations; inspired by the idea of welfare pluralism and human rights as self-determination and independent life, people with IDDs may be assisted in residential facilities or receive home and community-based care [8]. Under law 104/1992, an individual’s disability and related needs are assessed by a multi-disciplinary board of specialists. The evaluation is derived from a health model of disability and focused on assessing the degree of inability to work. Local units of the Italian National Health System (INHS) are the referral points and supervise the process, where the board compiles a diagnostic report informed by the ICD-10 guidelines from the World Health Organization.

In addition, individuals with IDDs in Italy receive a national cash allowance, designed to facilitate the direct purchase of care instead of directly provided care services. In practice, this cash allowance is used to integrate family income and is akin to a compensation for family members providing care [9]. While this cash-for-care scheme carries the risk of marketization and commodification of care services, people with IDDs and their families have been able to exercise freedom of choice, creating an incentive for providers to implement efficient and sustainable service solutions aligned with human rights requirements [10]. The INHS has been entrusted with the provision of habilitation services, either by directly establishing so-called social-rehabilitation centres or through accreditation and monitoring of private organizations. Supports are typically provided in the following:

- Day centres (for lower and higher support needs);
- Residential centres (for lower and higher support needs);
- Supports at home.

Fea noted that although each region in Italy has its own set of requirements over such centres, all have to conform to national law [11]. Services are required to meet the quality criteria of the funding institution and must be designed to support the person with disability wherever it seems necessary and reasonable [12]. Moreover, the INHS must guarantee the provision of a list of assistive technology and prosthetics supports that are included in the so-called “essential levels of care”. Italian support provisions for people with intellectual and developmental disabilities are framed by European policy through alignment with the UN Convention on the Rights of Persons with Disabilities (UNCRPD) and the EU Charter of Fundamental Rights, and are further guided by the EU’s Strategy for the Rights of Persons with Disabilities 2021–2030 and the Next Generation EU (NGEU) funds. The traditional Italian system has been called for renovation and paradigm change with Italy’s national reforms, such as the Law on Disability and the implementation of deinstitutionalisation projects using NGEU grants, which aim to strengthen community-based services and promote social inclusion in line with these European frameworks. The system is inspired by person-centred care with the aim to provide person-centred planning. Person-centred planning refers to a structured, collaborative approach to support provision that prioritizes the individual’s aspirations and strengths, with the overarching goal of enhancing community inclusion, meaningful relationships, self-determination, and personal competence [13,14,15]. Paradigmatic is the introduction of the policy reform by Legislative Decree n.62 in 2024. The recent reforms, particularly in disability services, focus heavily on personalizing support and empowering individuals to determine their own needs, a shift from previous models that largely centred around service providers’ offerings. This transformation places individuals at the heart of their own care and support planning, which aligns with global movements advocating for self-determination and independence for people with disabilities. The reform highlights the importance of tailoring services to individual preferences and needs, with an emphasis on the use of personal budgets. These budgets enable people with disabilities to directly control the financial resources allocated to their care, enabling more flexible and customized services.

The shift towards individualized services and the autonomy to make decisions about one’s support needs is crucial in fostering a sense of empowerment and improving life satisfaction. Research consistently shows that when people with disabilities can choose and manage their supports, it enhances their quality of life and participation in society [16,17]. This reform can be seen as a critical step towards making Italy’s disability policies more inclusive and responsive to the actual needs and aspirations of people with disabilities, moving away from the one-size-fits-all solutions. By shifting the focus from provider-driven budgets to user-controlled resources, the reform fosters a system where individuals can define their own life goals and determine the type and level of support they need [18].

This approach aligns with broader international trends in disability policy, including those seen in the UK, Sweden, and other European countries, where individualization and self-determination have been shown to significantly improve outcomes for people with disabilities [19,20,21,22]. Accordingly, this study aims to examine how socio-ecological factors influence resource allocation in person-centred case management for adults with intellectual and developmental disabilities (IDDs), and to assess the utility of a flexible multivariate modelling approach for capturing patterns of resource use in complex support provision.

## 2. Materials and Methods

This study employs a cross-sectional observational design, utilizing data from an experimental project funded by the Italian Ministry of Labor and Social Policy aimed at promoting social inclusion and quality of life (QOL) of adults with IDDs. The project had been instrumental to create ISPs for more than 1000 people with disabilities. Disability case managers composed the individual profile of service users and formulated a person-centred plan of supports aligned with individual needs. All case managers received an initial training in the best evidence-based practices aimed at improving quality of life [23] of people with IDDs, and were selected among experienced staff at the service centres.

Participants were recruited among clients of 23 ANFFAS NGO services in 13 Italian regions and selected by case managers to provide a representative sample of adults associated with their service. Data were collected between November 2014 and January 2015 in electronic format using the protocol developed by ANFFAS (Rome, Italy) “Matrici Ecologiche” (http://www.matriciecologiche.net)” (accessed 24 October, 2025, designed to support the creation of a person-centred plan within a QOL-oriented framework. The software aligns in a logical framework data collected from the assessment, with personal outcomes and supports to achieve the outcomes. It accounts the type and number of supports used to create an Individualized Support Plan in a person-centred way. In addition, an on-line community of practice was created, encompassing other service centres.

The analytic sample comprised 1152 adults with intellectual and developmental disabilities (IDDs) recruited from 23 ANFFAS NGO service centres across 13 Italian regions. Participants ranged in age from 16 to 88 years (M = 41.5, SD = 14.1), with 62% male (n = 705) and 38% female (n = 441). Levels of intellectual functioning were distributed as follows: mild (9%), moderate (24%), severe (26%), and no impairment reported (41%). Regarding living arrangements, 75% of participants resided with their families or independently, 12% lived in small residential facilities (<10 residents), and 13% in larger residential facilities (>10 residents). Approximately 31% were engaged in work, volunteering, or day centre activities.

Inclusion criteria required participants to be aged 16 years or older, formally registered with one of the participating service centres, and to have an Individualized Support Plan (ISP) developed by a trained case manager. Exclusion criteria were limited to incomplete records, particularly missing values on key socio-demographic variables or Supports Intensity Scale scores, which resulted in the final analytic sample of 1152 individuals [19]. ANFFAS head office collected the information and provided data anonymized to researchers. Case managers recorded personal characteristics, environmental variables, and formulated support plans framed within a person-centred approach and the QOL paradigm [23,24].

We measured resource use within ISPs by the weekly number of provided supports. We categorized supports and services according to the classification proposed in Claes, Van Hove, Vandevelde, van Loon, and Schalock [14] and in Lombardi et al. [25]:

- Community participation supports (COM.PART) intend to facilitate social integration;
- Adaptive skills supports (AD.SKILLS) develop positive behavioural skills and motivate clients toward capability and knowledge acquisition;
- Habilitation professional services (HABIL) address specific functional needs;
- PROSTHETICS enable and facilitate sensory–motor functioning;
- Technology (TECH) includes assistive and information technology to enhance cognitive functioning.

The key explanatory variables included the client’s intensity of support needs, extraordinary behavioural and medical support needs, and level of intellectual functioning.The Supports Intensity Scale (SIS; [26]) used in this study was the Italian adaptation validated by Leoni and Croce in 2008 and further tested for measurement invariance and reliability in Italian populations by Shaw et al. in 2022 [27,28]. The instrument has demonstrated strong internal consistency (Cronbach’s α ranging between 0.89 and 0.96 across domains) and robust construct validity for adults with intellectual and developmental disabilities. Although the SIS is standardized for adult respondents, our sample included a wide age range (16–88 years). While the tool is considered appropriate for adults across the life span, potential differences in how younger and older adults interpret or experience support needs were acknowledged when interpreting results. The SIS assesses the supports needed to participate in typical activities within ordinary environments, including home and community living, lifelong learning, employment, health and social activities, health and safety, as well as protection and advocacy. Case managers also recorded scores for extraordinary BEHAVIOURAL and MEDICAL needs. Level of intellectual functioning was collected from client’s INHS files.

Remaining control variables included client’s characteristics, family and client desires for improvement, living arrangement, and working status. All regression models controlled for the number of QOL domains represented among the desires and goals for improvement were expressed as priorities in ISPs by clients and their families. Recorded information also included living arrangements:

- independent;
- with family;
- residential service with <10 clients;
- residential service with >10 clients;

and working status:

- no activity;
- paid job;
- volunteering;
- day care centre activities.

The analysis was conducted using multivariate count regression models to account for the joint determination of different types of supports included in Individualized Support Plans (ISPs). We first fitted univariate models (Poisson, negative binomial, and Poisson-lognormal) to each support category—adaptive skills training, community participation, and habilitation services—selecting the best-fitting models based on Bayesian Information Criterion (BIC). These marginals were then combined within the Sarmanov–Lee multivariate framework, which allows flexible modelling of correlations and heterogeneity across support types. This approach enabled us to identify associations among supports while controlling for socio-ecological variables such as support intensity, living arrangements, working status, and assistive technologies. The methodology ensured that the analysis captured the interdependence of supports, providing a more accurate understanding of resource allocation in complex support provision contexts.

Table 1 shows definition and summary statistics of variables used in the empirical analysis. Composition by gender is displayed, but excluded from the reported results because it did not show a significant impact in the analysis. Mean number of AD.SKILLS in ISPs was 4.1 (SD = 2.61), while clients received 1.02 (SD = 1.09) HABIL services on average. In addition, the average counts of COM.PART supports, PROSTHETICS, and assistive TECH were 1.65 (SD = 1.65), 0.25 (SD = 0.57), and 1.62 (SD = 1.37), respectively. Interestingly, counts of adaptive skills training and community participation are overdispersed, while counts of habilitation interventions, prosthetics, and assistive technology are roughly equidispersed.

Information on the level of impairment of intellectual functioning was used to construct three dummy variables: MILD (n = 98, 8.51%), MODERATE (n = 273, 23.70%), and SEVERE (n = 303, 26.30%), with “no impairment” (n = 478, 41.49%) as the reference category. Support needs of the participants were on average in the 63rd percentile (SD = 16.4) as measured with the SIS. With regard to exceptional support needs, the participants displayed on average 3.66 (SD = 4.04) BEHAVIOUR and 1.63 (SD = 2.52) MEDICAL as measured by the respective sub-scales. The participants expressed on average DESIRES in 6.53 (SD = 2.41) out of 8 domains of QOL. Additionally, 31% of participants were involved in some forms of WORKING activities.

Those residing in SMALL residential facilities were 12%, and a similar percentage resided in LARGE contexts (13%), while the majority (75%) lived with their families or independently.

## 3. Econometric Framework and Model Selection

Given the features of the case management process, we considered the use of supports to be jointly determined. We employed a flexible multivariate count model allowing for positive, negative, or no association among the counts. Specially, we adopted the approach by Sarmanov and Lee to obtain multivariate distributions by given marginal [29,30]. Miravete showed that the Sarmanov model avoids overestimating the degree of association between uncorrelated count variables in small samples [31]. A valuable feature of the Sarmanov–Lee count models is that correlation and unobserved heterogeneity depend on different parameters, allowing for overdispersion and equidispersion (or underdispersion) in the marginals [30]. We took the following steps to find a good multivariate model of the form of the second equation: First, we fitted univariate models for each of the support counts and selected the best marginal models based on information criteria. Then, we estimated Sarmanov–Lee multivariate models combining maintained marginals and tested for association among the counts. Finally, we evaluated overall model specification through conditional moments tests.

Considering that the Poisson regression model (PRM) can be nested within the three mixed Poisson alternatives, namely negative binomial-1 (NB1), negative bino-mial-2 (NB2), and Poisson-lognormal (PLN), while the other models are not nested, we computed the Bayesian Information Criteria (BIC) to identify the best marginal model. Quasi-maximum likelihood (QML) and numerical optimization routines were employed for all the estimation procedures.

For two-part models, we assumed independence between the hurdle and the positive component, so the log-likelihood can be factored into two terms and separately maximized. When the likelihood of the mixed Poisson model required the evaluation of an integral, this was performed with Gauss–Hermite quadrature.

Our specific-to-general modelling approach calls for a rigorous evaluation of the maintained model. The goodness of fit of the multivariate models was assessed through conditional moment comparisons as developed in Andrews [32].

## 4. Empirical Results

### 4.1. Marginal Model Selection

Modelling multivariate count data with Sarmanov–Lee distributions enables the selection of models separately for each marginal. For counts of AD.SKILLS and COM.PART, it was possible to estimate one-part and two-part regression models for over-dispersed data, whereas attempts to fit mixing models for HABIL resulted in the convergence of numerical routines to the baseline PRM and hurdle probit zero-truncated Poisson. We used the Bayesian Information Criterion (BIC) to select the best marginal models. These are the negative binomial-1 for AD.SKILLS, the hurdle probit zero-truncated Poisson-lognormal (PLN) for COM.PART, while the standard Poisson regression model is enough for modelling HABIL, given the explanatory variables. We conducted the flexible multivariate count distributions analysis adopting the three above selected count data regression models.

### 4.2. Tests of Independence and Goodness of Fit of Multivariate Models

Computing Kendall’s τ statistics to evaluate the degree of dependence between the key count variables yielded all positive and statistically significant results, i.e., τ(AD. SKILLS, COM. PART) = 0.1154, z = 6.30, τ(AD. SKILLS, HABIL) = 0.1430, z = 7.74, τ(COM. PART, HABIL) = 0.0676, z = 3.72. We computed standard errors for z statistics with *somersd* Stata 16 package. The association was strongest between AD.SKILLS and HABIL, while COM.PART and HABIL showed the weakest degree of association. These results suggested that AD.SKILLS, COM.PART, and HABIL supports are considered functional complements in the socio-ecological logic of person-centred case management. Nonetheless, the detected bivariate associations in our sample are merely unconditional in nature and, for instance, cannot control for observed heterogeneity.

Table 2 displays estimates of association coefficients and goodness-of-fit measures for the multivariate Sarmanov–Lee models. Where bivariate association is allowed for only one pair of count variables, bivariate models 1–3, all three estimated coefficients are positive, with the association between COM.PART and HABIL significant at 1% level, and the other two models capturing associations at 5% significance level. The above results establish positive pairwise associations between the three counts.

Table 2 also reports the chi-square goodness-of-fit statistics calculated for the bivariate and trivariate Sarmanov–Lee count regression models. While all three bivariate regression models do not pass the goodness-of-fit test, the hypotheses that the two trivariate models are correctly specified cannot be rejected at standard significance levels. Taking into consideration these results and the statistical significance of the third order association coefficient, $\omega_{123}$, we adopted the trivariate model with full Sarmanov–Lee dependence structure for the computation of the marginal effects.

### 4.3. Marginal Effects

In this section, we present results based on the trivariate regression model with full Sarmanov–Lee dependence structure; this is the model denoted Trivariate Model 2 in Table 2. First, we discuss the age utilization gradient and marginal effects at the mean for prosthetics, assistive technologies, working status, and type of living arrangements. Then, results are presented for the supports intensity need utilization gradient and marginal effects of levels of intellectual functioning, exceptional behavioural and medical support needs, and expressed desires of the clients and their families.

**Figure 1:**
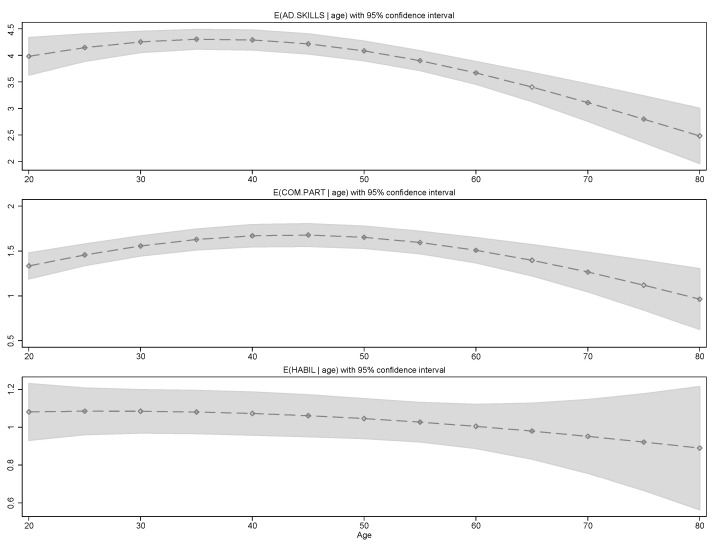
Expected number of supports included in Individual Support Plans (ISPs) as a function of client age. Grey color represents the 95% confidence interval.

Figure 1 illustrates the expected number of supports included in the ISPs as a function of the age of the client. AD.SKILLS training is provided consistently to younger clients, but we found a negative utilization gradient for middle-age and older clients. COM.PART has also a marked non-monotonic relation with age; the expected supports in this area reach a maximum for individuals aged 45 years. Interestingly, resource use on HABIL supports is not related to age in our model, once we control for a measure of support needs; to find otherwise could expose an “illusion of necessity” [33].

Table 3 shows that the presence of prosthetics has a positive impact on the inclusion of supports in ISPs. Compared to no prosthetics, having one PROSTHETIC support is associated with 0.71, 0.42, and 0.2 more supports in AD.SKILLS, COM.PART, and HABIL, respectively. Similarly, having one assistive technology device is associated with 0.41 and 0.1 more supports in AD.SKILLS and HABIL, while there is no significant impact on COM.PART. Working status only has direct influence on the number of AD.SKILLS training. Being involved in productive activities either inside or outside of the service centres is associated with 1.03 additional supports in adaptive skills.

Finally, results show that clients living in residential contexts have more supports included in their ISPs over all three categories, compared to clients living with families or independently. Living in small residential contexts (SMALL) is associated with 1.29 more AD.SKILLS, 0.35 more COM.PART, and 0.57 more HABIL. Similarly, living in larger residential contexts (LARGE) implies 1.51 more AD.SKILLS, 0.49 more COM.PART, but only 0.21 more HABIL.

**Figure 2:**
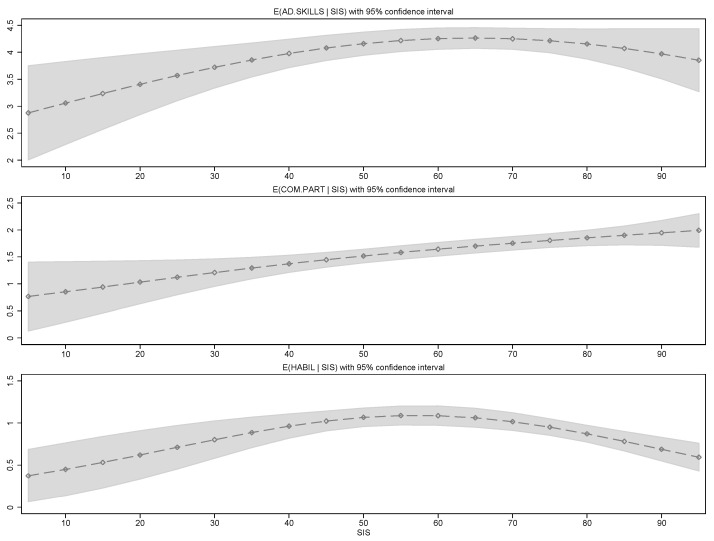
Expected number of support measures included in Individual Support Plans (ISPs) as a function of client’s SIS score. Grey color represents the 95% confidence interval.

The three panels in Figure 2 show the expected number of supports included in the ISPs as a function of the SIS percentile. The relationship between the number of AD.SKILLS and intensity of support needs is non-monotonic. Clients near the 65th percentile of the SIS measurement receive the highest number of adaptive skills training. Strikingly, individuals with the most intense needs do not receive more supports in this area. COM.PART shows a monotone relationship with SIS; clients with low levels of support needs see around 0.8 community participation supports in their ISPs, while clients in the higher spectrum of support intensity needs on average see 2.1 COM.PART included in their ISP. The number of HABIL supports are at their highest for clients in the 55th to 60th SIS percentile. Again, somewhat surprisingly, individuals in the top percentiles of the SIS measurement receive significantly fewer supports in this area.

Inspecting the marginal effects of intellectual functioning, reported in Table 3, clients with MODERATE impaired intellectual functioning receive 0.39 more COM.PART. Individuals with SEVERE intellectual impairment have 0.33 more AD.SKILLS and COM.PART included in their ISPs. For participants with MILD intellectual impairment, the joint model suggests no significant differences with the comparison group (no intellectual disability reported). Clients with extraordinary needs related to their BEHAVIOUR receive on average fewer AD.SKILLS and COM.PART supports, but their ISPs include more HABIL services. While extraordinary MEDICAL needs are not related to the composition of ISPs, this result arises once unobserved heterogeneity is allowed into the model. The number of DESIRES for improvements in the QOL domains when expressed by the clients and their families are positively associated with the average number of AD.SKILLS and HABIL supports, while they are negatively related to the counts of COM.PART supports.

## 5. Discussion

This study aimed to evaluate public policy outcomes in terms of services and supports provided and determine the social return on investment of social enterprises investigating how socio-ecological factors shape resource allocation in person-centred case management for adults with intellectual and developmental disabilities [4,34,35]. The data were collected in 2015 and are discussed in the context of the ongoing reform of Italy’s national support system. Thus, this study offers relevant evidence on how the traditional system of support allocation operated in sight of the current paradigm shift towards a human rights and QOL outcomes driven system. Given the study’s cross-sectional observational design, the findings provide a snapshot of the traditional system of support allocation at a specific point in time, offering valuable insights but also reflecting the inherent limitations of such a design, particularly in establishing causal relationships or tracking longitudinal trends.

The findings reveal several important patterns. First, there is a non-monotonic relationship between resources allocated in Individualized Support Plans (ISPs) and measured support intensity needs, indicating that individuals with intermediate levels of need tend to receive more supports than those with higher or lower levels of need. Second, person-centred support planning does not discriminate by gender or intellectual functioning once needs and contextual factors are controlled. Third, individuals in residential settings receive, on average, more supports—particularly oriented toward adaptive skills and habilitation within the service context—than toward community participation. Finally, the multivariate model accurately predicts how resources are distributed across community participation, adaptive skills training, and habilitation services.

These results align with international research linking QOL to support provision [14,19,36,37]. However, the purposive sampling approach introduces important considerations. While the sample was designed to reflect the diversity of service users, it was not randomly or statistically stratified. This approach may limit the generalizability of the findings, as the sample could overrepresent individuals whose needs align with the priorities or capacities of the participating centres. Importantly, the findings suggest that current systems may inadequately serve the most vulnerable individuals—those with the highest support needs—while focusing disproportionately on clients with average levels of need. This imbalance raises concerns about equity and effectiveness, particularly given that individuals with extraordinary behavioural needs appear to receive fewer supports for adaptive skills and community participation, even though their ISPs include more habilitation-oriented interventions. As Brady et al. observed, such gaps risk leaving the most vulnerable individuals, often with limited communication skills, underserved [38]. Conversely, clients with lower support needs may also be underserved in community-oriented supports, leaving them at risk of segregation [39]. Together, these findings indicate that although person-centred frameworks are being implemented, they still reflect traditional habilitation-oriented models rather than fully inclusive, socio-ecological approaches.

Interestingly, the finding that clients living with their families account for fewer supports may be explained by the tendency to include only formal supports in ISPs. This organizational bias could contribute to a system that remains service-centred rather than truly person-centred [40]. Consistent with a socio-ecological view, effective planning should sequence interventions from personal priorities to environmental and system accommodations, reflecting evidence that QOL applications are context-dependent and rights-anchored rather than service-centric [20,25].

Client-level implications.

Persons with disabilities, families, and case managers should incorporate a structured equity review into ISP development. In particular, plans for people in the upper decile of support intensity or with extraordinary behavioural needs should demonstrate balanced allocations across adaptive skills, community participation, and habilitation supports. For instance, if a plan emphasizes habilitation due to behavioural concerns, it should still include at least one community-participation activity to prevent social isolation. Given the negative age gradient in adaptive skills supports, reductions in training for middle-aged and older adults should be explicitly justified. It is essential to emphasize that individuals with disabilities (e.g., intellectual or motor) should actively cultivate their abilities, as skill development can progress over time regardless of impairment [41]. Early assistive technology reviews are recommended, as prosthetics and technology supports in our data were associated with richer, more enabling support packages.

Provider-level implications.

Provider organizations should formalize a community–connector function responsible for brokering inclusive roles, transportation solutions, and natural supports. In residential settings, rebalancing from service-internal habilitation toward externally oriented participation goals is warranted. Standard workflows should include assistive technology screening, environmental and community asset mapping, and transport/access supports before defining time-limited skill-building interventions, followed by a planned fading of personnel-driven supports where feasible. This perspective emphasizes naturally available supports and contextual facilitators that foster human functioning and participation in community life as any other citizen, not as a “disabled person.” The ultimate goal should be to build environments that are universally accessible [42,43].

System-level implications.

At the system level, organizations and policymakers should adopt case-mix-adjusted, outcomes-linked financing mechanisms supported by transparent equity audits. The literature has shown that data analysis and integration can help identify predictors, mediators, and moderators of QOL outcomes [19,24,44]. The present findings demonstrate that using data-driven approaches—such as our multivariate model to establish expected ranges for adaptive skills, community participation, and habilitation supports—can assist in identifying inequities in service provision. This reinforces an evidence- and value-based perspective: there is a need to operationalize human rights, foster full citizenship and inclusion, and employ evidence-based supports to achieve those outcomes [45]. Accordingly, contracting standards should include minimum inclusion criteria (e.g., at least one community-participation support per plan) and public reporting of a Support Equity Index by support needs tier and living arrangement. The Friuli Venezia Giulia region in Italy provides a relevant example of reform, where individualized project budgets are tied to personal goals rather than fixed service types (Regional Law 22/2019; Regional Law 16/2022). Such systems shift responsibility toward co-designed, outcome-oriented supports, allowing accredited organizations to manage flexible budgets that reflect real needs rather than standardized allocations. This approach highlights the potential of person-centred, data-informed models to replace service-driven systems and foster innovation in community-based supports.

## 6. Limitations

Nevertheless, the study is not without limitations. First, the cross-sectional design precludes causal inferences or the examination of longitudinal trends. Second, the participating organizations were not randomly selected, but were part of one single network of support providers (ANFFAS), and inclusion was voluntary. This may have introduced bias, as organizations more committed to self-assessment and outcome measurement were overrepresented, potentially limiting generalizability. Additionally, participant selection by case managers may affect both internal and external validity. Third, many organizations were unfamiliar with QOL and socio-ecological frameworks of support, leading to a predominance of staff-based supports in ISPs (particularly in adaptive skills training). This may reflect providers’ current orientations more than actual needs, and constrains the conclusions that can be drawn about the breadth of support provision. Fourth, a further limitation concerns the time frame of data collection (2014–2015), which preceded recent national disability reforms, including Legislative Decree n.62/2024 and the implementation of the Next Generation EU (NGEU) framework. Although the age of the data may limit direct comparability with current service models, they provide an important historical baseline for understanding how person-centred principles were operationalized before the policy transition toward individualized budgets and community inclusion. Future research using post-reform datasets will be crucial to evaluate whether these systemic changes have effectively addressed the equity gaps observed in this study. Future research should include longitudinal and experimental designs comparing standard and innovative support models to identify practices that most effectively promote personal outcomes [24].

## 7. Conclusions

In conclusion, understanding the interaction between contextual factors, support provision, and client outcomes is essential for effective and equitable systems [46]. At the micro level, person-centred case management should ensure individualized supports aligned with personal goals and participation in community life [25]. At the meso level, organizations and service providers have the opportunity to use evidence-based algorithms and flexible funding to match supports to actual needs while minimizing inequalities. At the macro level, policymakers should prioritize sustainable, data-driven systems that integrate personal and community resources. Taken together, the findings of this study, while subject to limitations, offer guidance for reducing discrepancies between assessed needs and provided supports, and for fostering collaborative, equitable, and innovative support ecosystems.

## Figures and Tables

**Table 1:** Summary of Individual Characteristics.

Variable	Mean	SD	Maximum
Supports count			
Adaptive skills	4.10 (4.43) ^a^	2.61	14
Community participation	1.65 (19.44) ^a^	1.65	13
Habilitation	1.02 (36.28) ^a^	1.09	7
Prosthetic supports	0.25 (80.90) ^a^	0.57	4
Technology-based supports	1.62 (23.09) ^a^	1.37	7
Female	0.38	0.49	
Age	41.5	14.1	
Intellectual disability			
Mild	0.09	0.28	
Moderate	0.24	0.43	
Severe	0.26	0.44	
SIS percentile	63.4	16.4	
Specialized support need scale			
Behavioural	3.66	4.04	
Medical	1.63	2.52	
QOL desires and expectations	6.53	2.41	
Working	0.31	0.46	
Residential context			
Small (<10)	0.12	0.33	
Large (>10)	0.13	0.34	

Note. n = 1152; SIS = Supports Intensity Scale percentile; QOL = quality of life ^a^ Frequency of zero counts.

**Table 2:** Estimates of association coefficients and goodness-of-fit measures for the multivariate Sarmanov–Lee models.

Association Coefficient	Bivariate Model 1	Bivariate Model 2	Bivariate Model 3	Trivariate Model 1	Trivariate Model 2
$\omega_{12}$	1.0418 *			1.2357 **	1.2887 *
$\omega_{13}$	(0.4619)	0.7999 *		(0.4738) 0.9630 **	(0.5329) 0.8817 **
$\omega_{23}$		(0.3737)	0.7493 **	(0.3647) 0.7861 **	(0.3392) 0.7254 **
$\omega_{123}$			(0.2564)	(0.2556)	(0.2568) −3.2026 *
					(1.4364)
$\chi_{CM}^{2}$	69.823 ***	49.021 ***	43.852 ***	28.78	28.263
df	17	16	12	28	28

Note: $y_{1}$: AD.SKILLS, $y_{2}$: COM.PART, $y_{3}$: HABIL; $\chi_{CM}^{2}$ = goodness-of-fit test; df = degrees of freedom of the goodness-of-fit test; QML robust standard errors in parentheses. * *p* < 0.05. ** *p* < 0.01. *** *p* < 0.001.

**Table 3:** Marginal effects on the expected number of supports included in the ISPs.

		Supports in ISPs	
Variable	Adaptive Skills	Community Participation	Habilitation
PROSTHETICS ^a^	0.709 *** (0.120)	0.422 *** (0.093)	0.200 *** (0.055)
TECH ^a^	0.409 *** (0.045)	0.004 (0.032)	0.101 *** (0.020)
BEHAVIOUR	−0.059 ** (0.019)	−0.026 * (0.011)	0.033 *** (0.007)
MEDICAL	−0.036 (0.029)	−0.055 ^†^ (0.031)	−0.004 (0.020)
*Level of Intellectual Disability*			
None	Ref.	Ref.	Ref.
MILD	−0.338 (0.251)	0.190 (0.137)	−0.084 (0.122)
MODERATE	0.288 (0.182)	0.395 *** (0.115)	−0.056 (0.082)
SEVERE	0.333 ^†^ (0.179)	0.331 ** (0.103)	−0.054 (0.076)
DESIRES ^b^	0.200 *** (0.034)	0.121 *** (0.020)	0.040 ** (0.013)
*Working status*			
Not working	Ref.	Ref.	Ref.
WORKING	1.033 *** (0.188)	0.110 (0.086)	0.011 (0.078)
*Living arrangements*			
Living at home	Ref.	Ref.	Ref.
SMALL	1.292 *** (0.243)	0.354 * (0.143)	0.572 *** (0.102)
LARGE	1.510 *** (0.240)	0.489 ** (0.167)	0.211 * (0.090)

Notes: For continuous variables, the table reports the effect of a (unitary) change in the variable on the expected number of supports included in the ISPs. For each dummy variable, the table reports the effect of the dummy variable going from 0 to 1 on the expected number of supports included in the ISPs. Standard errors in parentheses, computed with 200 draws from the covariance matrix of QML estimator. ^†^ *p* < 0.10, * *p* < 0.05, ** *p* < 0.01, *** *p* < 0.001. ^a^ Discrete change effect, one unit increase compared to zero count of supports ^b^ Considered as a continuous variable.

## Data Availability

The data presented in this study are available on request from the corresponding author due to personal and health information that does not allow public availability for privacy reasons.
